# Topological analysis as a tool for detection of abnormalities in protein–protein interaction data

**DOI:** 10.1093/bioinformatics/btac440

**Published:** 2022-06-30

**Authors:** Alicja W Nowakowska, Malgorzata Kotulska

**Affiliations:** Department of Biomedical Engineering, Faculty of Fundamental Problems of Technology, Wrocław University of Science and Technology, Wrocław 50-370, Poland; Department of Biomedical Engineering, Faculty of Fundamental Problems of Technology, Wrocław University of Science and Technology, Wrocław 50-370, Poland

## Abstract

**Motivation:**

Protein–protein interaction datasets, which can be modeled as networks, constitute an essential layer in multi-omics approach to biomedical knowledge. This representation gives insight into molecular pathways, help to uncover novel potential drug targets or predict a therapy outcome. Nevertheless, the data that constitute such systems are frequently incomplete, error-prone and biased by scientific trends. Implementation of methods for detection of such shortcomings could improve protein–protein interaction data analysis.

**Results:**

We performed topological analysis of three protein–protein interaction networks (PPINs) from IntAct Molecular Database, regarding cancer, Parkinson’s disease (two most common subjects in PPINs analysis) and Human Reference Interactome. The data collections were shown to be often biased by scientific interests, which highly impact the networks structure. This may obscure correct systematic biological interpretation of the protein–protein interactions and limit their application potential. As a solution to this problem, we propose a set of topological methods for the bias detection, which performed in the first step provides more objective biological conclusions regarding protein–protein interactions and their multi-omics consequences.

**Availability and implementation:**

A user-friendly tool Extensive Tool for Network Analysis (ETNA) is available on https://github.com/AlicjaNowakowska/ETNA. The software includes a graphical Colab notebook: https://githubtocolab.com/AlicjaNowakowska/ETNA/blob/main/ETNAColab.ipynb.

**Contact:**

alicja.nowakowska@pwr.edu.pl or malgorzata.kotulska@pwr.edu.pl

**Supplementary information:**

Supplementary data are available at *Bioinformatics* online.

## 1 Introduction

In the era of the constant growth of biological information, data analysis techniques are a topic hotter than ever in life sciences. The question of how to analyze such data is the key for retrieval of nature’s secrets and development of the society. The mathematically driven studies e.g. applied to discovery of novel disease genes, prediction of therapy outcome or improvement of diagnosis show a promising potential of this approach.

One of the disciplines that generate a lot of data of such great importance is protein–protein interaction (PPI) studies that generate protein–protein interaction networks (PPIN). The core characteristic of proteins is their ability to interact with one another and also with other types of molecules, in order to carry out their function. PPINs, which hold this property, can be understood as mathematical objects consisting of nodes—proteins and links between them—interactions. Understanding such graphs is crucial for further investigation of complicated molecular mechanisms that stand behind them.

The workflow necessary for the investigation of PPINs is provided by theoretical studies of graphs and networks. Modern Network Science concentrates on topological analysis of systems and their dynamics (Ted, 2013) with a common theme regarding modeling real networks. Initial studies in this field, mostly based on the mathematical work of Erdös and Rényi (1961) in the 60s, were focused on approximating real networks using random graphs with a degree distribution close to the Poisson distribution. Nevertheless, such graphs failed to reproduce two essential characteristics of the real systems—their small-world property and highly heavy-tailed degree distribution. The first one was captured by the Watts–Strogatz model based on random network rewiring, which led to a higher clusterization and shorter paths lengths (Watts and Strogatz, 1998). Despite these accomplishments, the resulting degree distribution was still Poisson-like. A breakthrough in understanding and modeling of the real networks was made by Barabási, who proposed a model reproducing a scale-free structure. In the network context this property, observed across multiple disciplines, is exhibited by the existence of multiple low-degree nodes and a few hubs dominating the network. To account for this phenomenon, the Barabási–Albert model assumed that a network emergence is based on the so-called ‘preferential attachment’ concept. According to this idea, nodes with a higher degree have a higher probability of linking to new nodes, reflecting a ‘richer get richer’ postulation (Barabási and Márton, 2016).

Given high complexity of biological systems, their analysis is hard to be performed with conventional tools. Therefore, research in disease comprehension and treatment development may hugely benefit from a network-based modeling. Identification of the most important proteins in a system, obtained with topological measures, could give a list of potentially best drug targets. Such a study was, e.g. performed by Rakshit *et al.* (2014), where basic centrality parameters were applied to study a PPIN related to the Parkinson’s disease. As a result, 37 novel molecules were associated to be highly related to this disorder. Moreover, enrichment of the data with bioinformatics information about molecular functions of the proteins could unravel details of molecular processes, which are disturbed in a state of the disease. This is the case for Wu *et al.* (2010) where PPINs for breast, colorectal and pancreatic cancers were created and compared, suggesting common mechanisms taking place in these disorders. Finally, it is even possible to try to predict new protein characteristics or functions on the basis of its network location, by combining the network analysis with other computational tools (Safari-Alighiarloo *et al*., 2014, Vazquez *et al*., 2003). The main focus in the PPIN literature is given to cancer, neurodegenerative disorders and virus infections—prominent examples of complex issues requiring innovative approaches and ideas.

Although the above mentioned studies already demonstrate the power of the method, it should be mentioned that network studies of PPI systems have several limitations. It is often not taken into account that proteins create a dynamic system in which the interactions happen within time and have a temporal character. In reality, a single protein only interacts with a subset of its possible partners at a specific moment. Despite that, in a common static network model each protein has permanent links with all its possible partners. Even more importantly, the studies of PPINs are varied in terms of data gathering procedures. Different detection methods of the protein interactions are known, each of them having its pros and cons, and all of them being error-prone and leading to some false positive results (Berggård *et al.*, 2007; Yugandhar *et al.*, 2019). Frequently, the networks are based on different sources of the data, which mix experimental techniques. In consequence, a heterogeneous structure of the data appears. Moreover, the initial choice of the proteins to be covered in the system is also crucial. It is often performed with certain bias resulting from a specific character of the study. Generalizations about the networks structures are necessary, although they are often based on networks obtained within one research group and the results generated by different techniques are not always included. Therefore, application of mathematical tools and topological measurements may improve the quality of such networks analysis.

In this study, we aimed to define a set of crucial measures and characteristics essential for PPINs evaluation. These methods are able to show possible biases and other issues with network structures that may influence biological interpretations of such systems. Finally, a topological analysis of different experimental human PPINs was performed, with an objective of examining variability and similarities in their structures, as well as finding possible intrinsic issues with their data. Given the IntAct popularity, we chose Cancer’s PPIN, Parkinson’s disease PPIN and Human Reference Interactome (HuRI) included in the IntAct resources. Finally, for those who may need to perform such analysis, we provide a user-friendly tool to exhaustively evaluate their networks quality based on multiple measures and simulations.

## 2 Materials and methods

### 2.1 Data

**Fig. 1.**
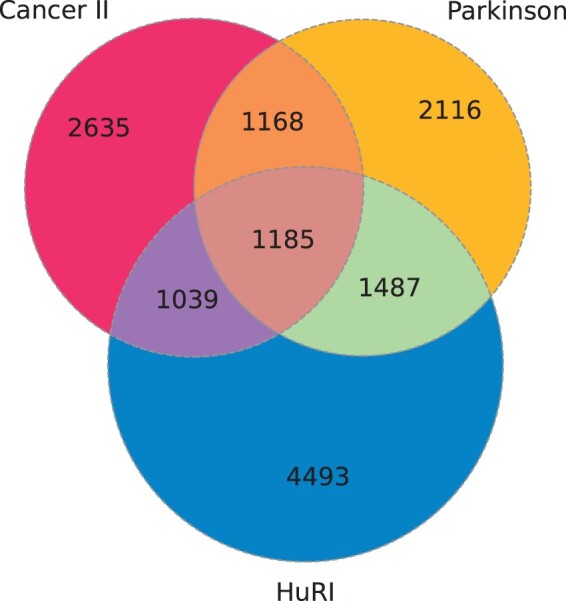
Venn diagram for proteins included in the datasets

**Fig. 2.**
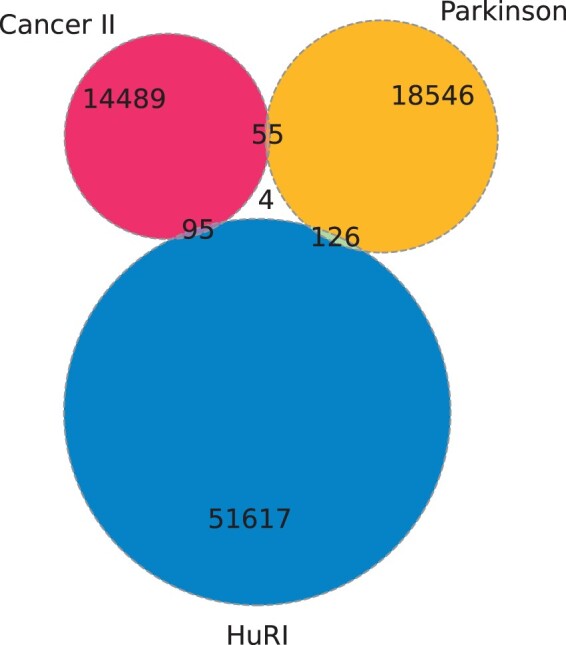
Venn diagram for unique interactions between proteins in the datasets

The three *IntAct* datasets were analyzed: two sets as manually curated datasets: *Parkinson* and *Cancer*, and *HuRI* (EMBL-EBI, 2021). The first dataset, *Parkinson*, created by the *IntAct* curators regards proteins and their interactions in the context of Parkinson’s disease. During the data gathering procedure, the particular focus was given to LRRK2 protein (Leucine-rich repeat kinase 2). This dataset was downloaded in two timestamps: November 4, 2020 and November 8, 2021. The original set from November 4, 2020 contained 59 912 links between 5955 proteins. The year after it was enriched by 22 new proteins and 70 interactions and it lost 21 proteins and 65 interactions, which gave, in total 55 930 links between 5956 proteins. Due to such minor changes, this network can be assumed to be structurally stable within time and only the newer version of the dataset is considered. The second dataset, *Cancer*, regards interactions of proteins that are involved in cancer. As previously, the data were also derived from a literature survey. This dataset was also downloaded in two timestamps: February 1, 2021 and November 8, 2021. In the first route (*Cancer I*) it contained 20 826 links regarding 5380 proteins and in the second (*Cancer II*) 23 263 links regarding 6027 proteins. *Cancer II* contains 2295 new interactions [with 1246 coming from Adhikari and Counter (2018), in which the focus was given to KRAS, HRAS and NRAS interactomes], and 658 new proteins compared to *Cancer I*. *Cancer II* has lost 75 interactions and 11 proteins present in *Cancer I*. Comparative analysis of these two sets may provide insight into temporal changes of such network characteristics. The third dataset, *HuRI*, is derived from one publication (Luck *et al.*, 2020). The set can be downloaded by providing the paper id *IM-25472* in the *IntAct* search box. These PPIs are estimated to cover 2–11% of all the interactions present in the human body. The proteins of this set were selected since they correspond to the genes that are robustly confirmed to be expressed in humans. The original set contained 162 719 links between 8204 proteins. Two Venn diagrams for *Parkinson*, *Cancer II* and *HuRI* were generated to compare the overlaps between the sets of proteins (Fig. 1) and the sets of links—interactions between the proteins included in each dataset (Fig. 2). Some protein groups are shared between the datasets, but sets of interactions are very different (some are not unique). If any of the datasets was complete, it should cover interactions discovered in other studies as well, which is not the case here.

The datasets’ records have *IntAct MI scores* indicating their reliability. We used them to assess the quality of the datasets interactions.

### 2.2 Theoretical methods

Based on the datasets of choice, we generated PPINs understood as networks *G*(*V*, *E*), where *V* is a set of vertices (nodes) and *E* is a set of edges (links). To prepare the PPINs for the analysis of their topological features, we removed the parallel links and all the networks components except the largest connected one, thus updating the *V* and *E* sets. The number of nodes (here: proteins) and the number of links between the nodes in the network (here: protein interactions) are denoted as N=|V| and L=|E|, respectively.

Degree of a node *k* is defined as a number of its neighbors, which is equivalent to the number of protein’s interactors. Nodes of a high degree are termed hubs. PPINs and other real networks tend to show scale-free property (Barabási and Márton, 2016; Yook *et al.*, 2004), in which the degree distribution (*p_k_*) follows a power law $p_{k}∼k^{-\gamma }$. Fitting of *γ* was performed using a maximum likelihood estimator since this method was shown to be the most effective (Clauset *et al.*, 2009). The fitting procedure is described in the SupplementarySectionS3.3. The value of *γ* is expected to belong to the interval (2, 3) (Barabási and Márton, 2016). Its value above three indicates that hubs in the network have a lower degree and hence are less impactful than if in the expected interval. The values below two are strongly anomalous.

Degree correlation coefficient is defined as (Newman, 2003):

(1) $$r=\frac{\sum_{x}e_{xx}-\sum_{x}a_{x}b_{x}}{1-\sum_{x}a_{x}b_{x}},$$

where *e_xy_* denotes a fraction of links in the network that connect nodes of degree *x* with nodes of degree *y*, $\sum_{x,y}e_{xy}=1$ and $\sum_{y}e_{xy}=a_{x},\;\sum_{x}e_{xy}=b_{x}$. In undirected networks, as in our case, there is assumed to be two links between linked nodes (one in each direction). The value of *r* belongs to <−1,1>, where *r *<* *0 means that the network is *dissasortative* (nodes tend to link to nodes of dissimilar degree), *r *>* *0 means that the network is *assortative*, while *r *=* *0 corresponds to a random distribution of links between the nodes.

Average Nearest Neighbour Degree (ANND) for the *i*th node is defined as:

(2) $$a_{nn}(i)=\frac{1}{k_{i}}\sum_{j=1}^{N}A_{ij}k_{j},$$

where *A_ij_* takes value one if there is a link between the *i*th and the *j*th nodes and zero otherwise. *ANND plot* is a plot of *k* versus average $<a_{nn}(i)>$, where $i\in \{v:k_{i}=k\;\text{and}\;v\in V\}$. In normalized ANND, both *k* and $<a_{nn}(i)>$ are normalized with respect to *N*.

To study the impact of groups of nodes with respect to their degree, we define subnetworks $G_{K}(V_{K},E_{K}),\;K=1\ldots k_{\text{max}}$, such as:

(3) $$\begin{array}{c} V_{K}=\{v:k_{v}\leq K\;\text{and}\;v\in V\}\\ E_{K}=\{E_{ij}:\;i,j\in V_{K}\}. \end{array}$$

*N_K_* and *L_K_* are the numbers of nodes and links in the subnetworks, respectively. We propose two novel subnetwork characteristics, which can show a different contribution of low and high degree nodes to the total number of links in the original network:

(4) $$s_{1}(K)=\frac{L_{K}}{L},$$

(5) $$s_{2}(M_{K}=\frac{N_{K}}{N})=\frac{L_{K}}{L}.$$

Betweenness centrality for the *i*th node is defined as:

(6) $$B(i)=\sum_{k,l;\;k\neq l\neq i}\frac{\sigma_{k,l}(i)}{\sigma_{k,l}},$$

where $\sigma_{k,l}(i)$ is a number of shortest paths crossing through the *i*th node, linking nodes *k* and *l*; $\sigma_{k,l}$ is a total number of shortest paths linking nodes *k* and *l*, where length of the path is a number of links between a pair of nodes. Nodes with high *B* value are termed bottlenecks.

Closeness centrality assesses how far a node is located from other nodes. For the *i*th node it is defined as:

(7) $$C(i)=\frac{1}{\sum_{j,\;i\neq j}d(i,j)},$$

where *d*(*i*, *j*) denotes the length of the shortest path between *i*th and *j*th node.

Eigenvector centrality *E*(*i*) defines the *i*th node importance in terms of the importance of its neighbors. It is calculated as the *i*th element of the eigenvector, corresponding to the highest eigenvalue of the network adjacency matrix *A*. *A_ij_* =1 if there is a direct link between *i*th and *j*th nodes, and zero otherwise.

Clustering coefficient reveals density of links in the node neighborhood. For the *i*th node it is calculated as:

(8) $$C_{l}(i)=\frac{2L_{i}}{k_{i}(k_{i}-1)},$$

where *L_i_* represents the number of direct links between the neighbors of the *i*th node.

Network robustness can be examined with respect to targeted attacks. Such simulations are typically based on degree and betweenness centrality. The nodes are sorted according to the centrality measure and removed in a descending manner according to the metric values. In our study of targeted attacks, we also involved eigenvector centrality and closeness centrality. It is possible to calculate a fraction of nodes *d* that leads to complete network decomposition. In our study, *d_k_* and *d_B_* are the points where the relative largest component size is below 0.01 for the targeted attacks using degree and betweenness centrality, respectively. In addition, network robustness can be measured with respect to random failures (Barabási and Márton, 2016). Further details of both algorithms can be found in SupplementarySectionS3.4.

Failure cascade simulation evaluates the error propagation potential of each of the nodes, as well as the network robustness towards failure spread. The simulation is performed with respect to a parameter *F*. An initial node is provided and its status is changed to *failed*. In the next step, in an iterative process, each network node is chosen. If its status is *not failed* but fraction *F* of its neighbors have status *failed* it also changes to *failed*. If iterating over all nodes leads to at least one change to *failed* the loop is repeated. Finally, a percentage *P* of nodes with the status *failed* at the end of the simulation is returned. To the best of our knowledge this kind of simulation has not been performed for PPINs. The proposed algorithm of the simulation was inspired by Barabási and Márton (2016) and its details can be found in SupplementarySectionS3.5.

## 3 Computational tools

For the fast and extensive network’s analysis, the methods were implemented as Extensive Tool for Network Analysis (ETNA). ETNA is based on Python graph-tool library (Peixoto, 2020), which significantly reduces calculation time due to C++ data structures and algorithms. In consequence, even for huge PPINs, the results are obtained within, in maximum, a few minutes. Graph-tool was integrated with numpy, pandas, random, rpy2, ipywidgets, base64, hashlib and typing, also available in Python, and with poweRlaw library from R (Gillespie, 2014). The additional information on how to use ETNA can be found in the SupplementarySectionS2. The tool is available on GitHub: https://github.com/AlicjaNowakowska/ETNA. The software includes a graphical Colab notebook https://githubtocolab.com/AlicjaNowakowska/ETNA/blob/main/ETNAColab.ipynb.

## 4 Results

### 4.1 Datasets characterization

The following characteristics of the datasets were noted.

**The PPI datasets can be generated based on different assumptions**. The proteins included in *Cancer* and *Parkinson* were chosen as the ones characteristic of the disease, according to the Genome Wide Association Studies. The opposite concerns *HuRI*. This dataset only includes proteins significantly expressed in humans in general.

**Different experimental techniques could be used to construct one PPI dataset**. In *Parkinson*, 85% of the records come from Haenig *et al.* (2020), which used two-hybrid method. This technique is a dominating experimental method in the dataset regarding 85% of the records. Other 10% have an anti-tag coimmunoprecipitation label. The distribution of *IntAct MI score* (Supplementary Fig. S1) has two regions of concentration. Almost all the records in *HuRI* (99.7%) were obtained using yeast-two-hybrid methods. In consequence, the distribution of the *IntAct MI score* value is very concentrated. The rare outliers happened because some interactions were also confirmed by other studies increasing the score value. In *Cancer I*, 30% of the records come from a publication (Kennedy *et al.*, 2020), the rest has no dominating source. Therefore, 55% of the records have a coimmunoprecipitation tag, the rest of the techniques were diverse e.g. tandem affinity purification, pull-down, protein kinase assay etc. The *IntAct MI score* values are much lower than in the other datasets. In *Cancer II*, 52% of the records have the coimmunoprecipitation tag, and the second important group regarding 7% of the records are a proximity-dependent biotin identification. It appears due to the inclusion of multiple (1246) new records derived from one publication (Adhikari and Counter, 2018). As in the *Cancer I*, the rest of the techniques is diverse and the *IntAct MI score* values are similarly low.

**Records regarding non-proteins are present**. *IntAct* included also other possible classes e.g. *small molecule*, *gene*, *single/double stranded deoxyribonucleic acid*, *molecule set* and *peptide*. *Parkinson* had 34 items that were assigned classes other than *protein* and 20 of them were *small molecules*. *Cancer I* had 617 items that were assigned classes other than *protein* and 358 of them were *genes*. One item was unlabeled. *Cancer II* had 610 items that were assigned classes other than *protein* and 349 of them were *genes*. *HuRI* had three items belonging to the *molecule set* class. The non-protein items were not dominating in the datasets. They can have biological meaning e.g. ponatinib and imatinib, cancer drugs, were tyrosine kinase inhibitors present in *Parkinson*. Ponatinib was assigned two records linking it with LRRK2 node and imatinib was assigned two records linking it with LRRK2 node and another two with moesin node (Ray *et al.*, 2014).

**Non-human taxids are present**. Each protein in *IntAct* was assigned to its source taxid. Some of the proteins within IntAct exist in a few versions. For example, in *Parkinson* there is a Prkn (EBI-973635), which corresponds to parkin protein with a *Mus musculus* taxid. In addition, there also exists a PRKN (EBI-716346), which corresponds to parkin protein with a *Homo sapiens* taxid.

**Around half of the records are repeated**. Some repetitions occurred because certain specific information reported in the articles was lost in the database e.g. due to different protein variants. It means that multiple protein alternatives were analyzed in the experiment although all were saved under the same identifier in *IntAct*. For example, interactions between different oligomers of a-synuclein (SNCA in *IntAct*) were recorded as multiple self-loops for SNCA node (Roberts *et al.*, 2015) (interactions ids: EBI-10690046, EBI-10690676 and EBI-10690707). Another observed reason is that a discovered interaction between *Protein A* and *Protein B* happened to be recorded in the database as (*Protein A*, *Protein B*) and (*Protein B*, *Protein A*), which led to the repetition. The removal of repetitions led to a significant datasets size reduction: *Parkinson* had 18 731 records, *Cancer I* 12 423, *Cancer II* 14 643 and *HuRI* 51 842.

**Datasets contain disconnected parts**. Considering the datasets with removed records repetitions as PPINs, it is found that *Parkinson* had 17 *components* (isolated network parts) with the *largest connected component* containing 99.4% of all nodes and 99.8% of all links. The rest of the components had up to five nodes. *Cancer I* had 120 components with the largest connected component containing 93.4% of all nodes and 97.6% of all links. The rest of the components had up to 13 nodes. *Cancer II* had 115 components with the largest connected component containing 94.3% of all nodes and 98% of all links. *HuRI* had 71 components with the largest connected component containing 98.5% of all nodes and 99.8% of all links. The rest of the components had up to three members (Table 1).

**Table 1.** Summary of PPINs after processing (parallel links and disconnected parts removed)

PPIN	Final *N*	Final *L*	Mean IntAct MI score	Dominating detection method	Special feature
*Parkinson*	5920	18 703	0.52	Yeast-two-hybrid	Exceptional focus on one protein
*Cancer I*	5025	12 122	0.42	Coimmunoprecipitation	Lower reliability of links
*Cancer II*	5688	14 359	0.4	Coimmunoprecipitation	Lower reliability of links
*HuRI*	8082	51 758	0.58	Yeast-two-hybrid	General study

### 4.2 Topology of the PPINs of choice

**Degree distribution.** As expected, in all the networks, we observed heavy-tailed degree distributions with the majority of nodes having a low degree and few exceptional hubs (Supplementary Fig. S2). The relative biggest hub sizes were as follows: *Parkinson* $k_{\text{max}}/N=0.37$ (LRRK2), *Cancer I* $k_{\text{max}}/N=0.064$ (ESR1), *Cancer II* $k_{\text{max}}/N=0.082$ (NRAS) and *HuRI* $k_{\text{max}}/N=0.064$ (CYSRT1). The statistically significant adjustment of the power-law parameter *γ* (*P*-value¿0.1) to the networks’ degree distributions is summarized in Table 2. In *HuRI*, the fitting is problematic and can be done for a few nodes only.

**Table 2.** Results of power-law fitting

PPIN	$\hat{\gamma }$	Cutoff value	Fraction of nodes described by the power law (%)
*Parkinson*	2.2	10	11
*Cancer I*	2.16	4	26
*Cancer II*	2.21	5	20
*HuRI*	3.3	67	4

**Fig. 3.**
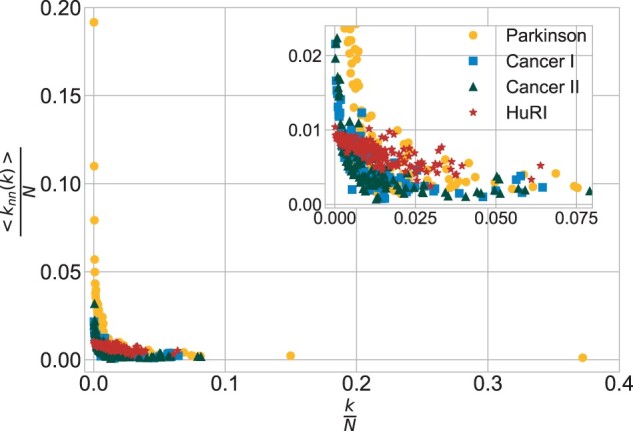
ANND plot for all networks

**Assortativity.** The degree correlation coefficients were as follows: *Parkinson* r=−0.018, *Cancer I* r=−0.001 and *Cancer II* r=−0.011. The ANND plots for these networks have hyperbolic-like scatterings. This indicates disassortative mixing patterns, common for PPINs. For HuRI *r *=* *0.008 and the ANND has a distinct scattering pattern (Fig. 3). This indicates a more assortative mixing pattern.

**Fig. 4.**
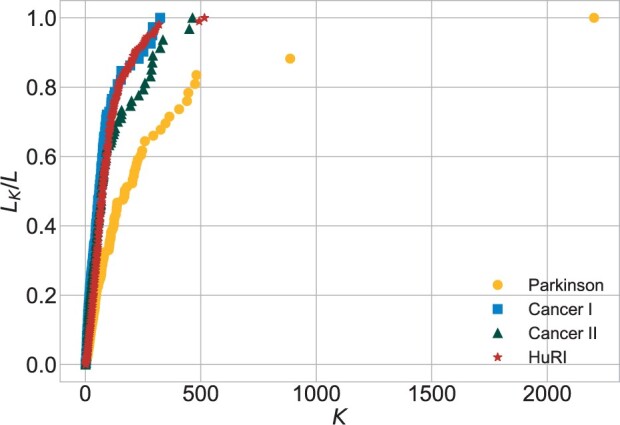
Impact of nodes with respect to their degree (*s*_1_)

**Fig. 5.**
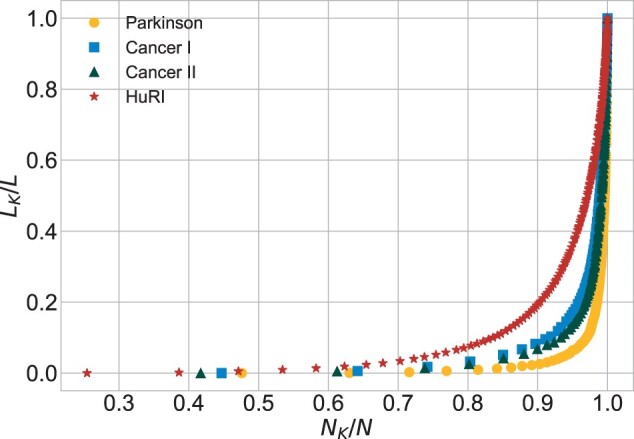
Impact of nodes with respect to their degree (*s*_2_)

**Impact of group of nodes with respect to their degree**. The sizes of the subsequent subnetworks were calculated in order to prepare the plots of *s*_1_ (Fig. 4) and *s*_2_ (Fig. 5). The two biggest hubs (LRRK2 and HTT) in *Parkinson* generate 17% of all the links. In this network, 90% of the nodes, all of them with k≤10, are responsible for 3% of all the links. In *Cancer I*, the two biggest hubs generate 5% of all the links and 91% of the nodes with k≤8 provide 9.5% of all the links. In *Cancer II*, the two biggest hubs generate 7% of all the links and 91% of the nodes with k≤8 provide 8% of all the links. In *Cancer II*, hubs are more impactful than in *Cancer I*. In *HuRI*, hubs contribution is not so significant. It was revealed by little jumps present in the plot of *s*_1_. The lowest degree nodes (*k *<* *34), which account for 90% of all the nodes, generate 20% of the links. In this network, significantly fewer nodes have *k *=* *1.

**Centrality measures**. The summary of the results for the betweenness centrality *B*, eigenvector centrality *E* and closeness centrality *C* is provided in Table 3, the metrics histograms are provided in Supplementary Figures S3–S5. In all PPINs, *B* and *E* have heavy-tailed distributions too. On the other hand, the *C* distribution is concentrated around its mean. LRRK2 in *Parkinson* is responsible for a much longer tail in the *B* and *E* distribution than it is for other PPINs of choice, similarly as in the degree analysis. However, the second largest values for these metrics in *Parkinson* are comparable to other PPINs. They belong to the HTT node—huntingtin protein ($B_{\text{HTT}}=0.16,\;E_{\text{HTT}}=0.23,\;C_{\text{HTT}}=0.47$). The generally higher values of *C* in *Parkinson* indicate smaller distances in this network. This is due to the focus on LRRK2, which shortens the lengths of the paths.

**Table 3.** Statistics for the centrality measures

PPIN	$B_{\text{max}}$	$E_{\text{max}}$	$C_{\text{max}}$	<C>
*Parkinson*	0.55 (LRRK2)	0.49 (LRRK2)	0.54	0.32
*Cancer I*	0.13 (EGFR)	0.26 (HSB1)	0.37	0.24
*Cancer II*	0.11 (EGFR)	0.43 (NRAS)	0.37	0.24
*HuRI*	0.09 (MEOX2)	0.22 (CYSTR1)	0.4	0.27

**Clustering coefficient**. The mean clustering coefficient equals as follows: *Parkinson* $<C_{l}>=0.1$, *Cancer I* $<C_{l}>=0.14$, *Cancer II* $<C_{l}>=0.19$ and *HuRI* $<C_{l}>=0.06$. The distribution of the metric has two regions of concentration for *Parkinson*, *Cancer I* and *Cancer II*. The first one is dominating and corresponds to the value of *C_l_* slightly above zero. The second region is much smaller and observed around 1.0. It indicates the existence of a very highly connected region(s) in these networks, which regards 6% (*Parkinson*), 8% (*Cancer I*) and 13% (*Cancer II*) of nodes. In *HuRI*, the second region with a high clustering coefficient value is barely noticeable. Histograms in Supplementary Figure S6.

**Fig. 6.**
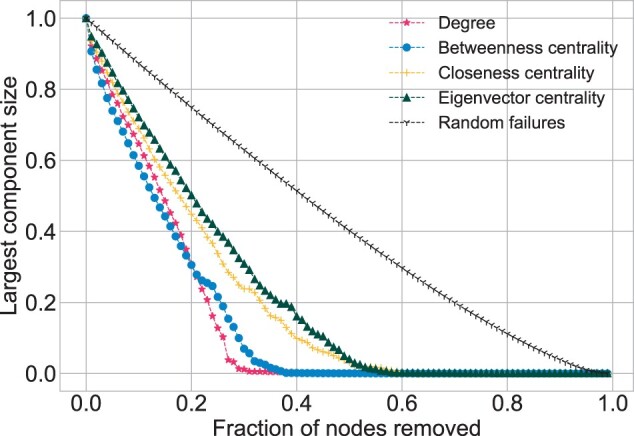
Robustness for *HuRI* network. Number of Monte Carlo simulations for random failures is MC* *=* *100

**Fig. 7.**
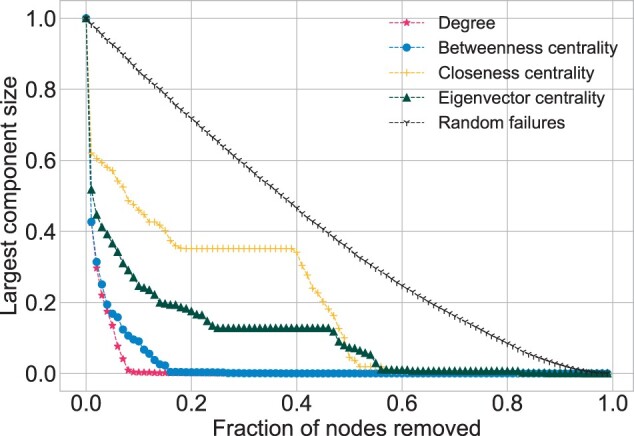
Robustness for *Parkinson* network. Number of Monte Carlo simulations for random failures is MC* *=* *100

**Robustness**. For all the networks similar results, in terms of random failures, were obtained. In the figures [*HuRI* (Fig. 6), *Parkinson* (Fig. 7), *Cancer I* and *Cancer II* (Supplementary Fig. S9)] the corresponding random failure scatterings have a linear trend, indicating high robustness to this kind of network decomposition, typical of all scale-free networks. Another observable result is separation of the scatterings into two groups. The first group belongs to degree and betweenness centrality, which led to the similar results. Degree always leads to a quicker decomposition except for the initial phase of the simulation in *HuRI*, in which the betweenness centrality is faster. The second group includes closeness and eigenvector centrality, which generate relatively similar scatterings in all the networks. In *HuRI*, all the scatterings have a similar shape, contrary to what is observed for *Parkinson*. In the latter network multiple jumps, especially for eigenvector and closeness centrality, are noticeable. *Cancer I* and *Cancer II* fall in between *HuRI* and *Parkinson* results. The decomposition fraction was as follows for the degree: *Parkinson* $d_{k}=0.08$, *Cancer I* $d_{k}=0.1$, *Cancer II* $d_{k}=0.09$ and *HuRI* $d_{k}=0.31$. For the betweenness centrality was as follows: *Parkinson* $d_{B}=0.16$, *Cancer I* $d_{B}=0.14$, *Cancer II* $d_{B}=0.15$ and *HuRI* $d_{B}=0.37$. Therefore, it can be stated that *Parkinson*, *Cancer I* and *Cancer II* are similarly vulnerable to the targeted attacks, in contrast to *HuRI*, which is much more immune.

**Failure cascade**. In all PPINs of choice, the failure cascade sizes have heavy-tailed distributions. The biggest values were obtained for *F *=* *0.25 and the smallest for *F *=* *0.75. The percentages of nodes capable of propagating the error are presented in Table 4 and the maximal cascade sizes in Table 5. The exceptionally powerful LAMP2 node in *Parkinson* for *F *=* *0.25 does not have very high values of any other centrality measures. HSBP1, MEOX2, NUDCD1, AR and RAVER1 nodes, which generate the maximal failure cascade sizes, are significant hubs and bottlenecks in their corresponding networks.

**Table 4.** Percentages of nodes capable of propagating an error

PPIN	*F *=* *0.25 (%)	*F *=* *0.5 (%)	*F *=* *0.75 (%)
*Parkinson*	10	5.7	3.5
*Cancer I*	23	15	9
*Cancer II*	20	13	8
*HuRI*	3	2	1

**Table 5.** Maximal failure cascade sizes ($P_{\text{max}}$)

PPIN	*F *=* *0.25	*F *=* *0.5	*F *=* *0.75
*Parkinson*	99% (LRRK2, LAMP2)	28% (LRRK2)	21% (LRRK2)
*Cancer I*	4% (NUDCD1)	3% (HSPB1)	2% (HSBP1)
*Cancer II*	29% (AR, RAVER1)	3% (HSPB1)	2% (HSBP1)
*HuRI*	2% (MEOX2)	1.5% (MEOX2)	1% (MEOX2)

## 5 Discussion

Using the qualitative and topological methods, implemented in our ETNA, we analyzed three different datasets regarding PPIs in humans. First, in the qualitative step, we assessed the datasets quality by examining the publications and experiments that provided the data. In the second topological step, we investigated the generated networks structures, applying a variety of classical and novel network science tools. Our study revealed an unexpectedly wide range of topological characteristics observed in PPINs. Some of them showed anomalies deviating from our current knowledge of biological networks. Our results suggest that unusual topological characteristics may appear due to a non-uniform interactome sampling since their appearance is clearly correlated with the data gathering procedure. We highlighted how each of the anomalies impacts on the network structure and which areas of biological interpretations may be affected. This knowledge can be applied by the users and authors of PPI datasets to assess the level of interest bias introduced in the data. When the topological characteristics are similar to these obtained in our study, a methodological bias in the data collection could be suspected. In such a case, the corresponding biological conclusions should be drawn with caution (see SupplementarySectionS4).

To account for the diversity of available PPIN studies, we studied a single experiment-based network, where a uniform sampling of the interactome was an important assumption of the workflow (*HuRI*) and literature-based meta-analysis datasets (*Cancer I*, *Cancer II* and *Parkinson*). However, *Parkinson* analysis revealed that 85% of the links came from a single experiment performed by Haenig *et al.* (2020). *Cancer II* was an updated version of *Cancer I*, which included more recently reported interactions resulting in 17% growth in the number of interactions and 10% in the number of proteins. Similarly as in *Parkinson*, half of the new interactions introduced in *Cancer II* came from a single experimental publication. The qualitative study revealed different levels of methodological heterogeneity of literature-based datasets. Multiple unexpected events, such as non-protein nodes, non-human taxids, temporal inclusion or removal of the interactions in the network evolution, parallel links, different qualities of the interactions within the networks and different experimental methods used, were observed. Regarding *HuRI*, multiple interactions and proteins were not included in this dataset, which can be noted when analyzing the Venn diagrams for PPINs. Such incompleteness of the data generated by yeast-two-hybrid screening is a common event. Even when analyzing the same yeast interactomes, using the same experimental method in each yeast-two-hybrid screen, highly different datasets can be produced (Ito *et al.*, 2001; Uetz *et al.*, 2000; Yook *et al.*, 2004).

The topological analysis revealed that the examined networks tend to demonstrate, on a general level, topological features typical of biological networks. In consequence, the datasets, at least partially, represent a corresponding biological phenomenon, as expected. Nevertheless, a more detailed analysis showed some discrepancies in their characteristics. This indicated locally important disturbances, which may lead to limited potential of the datasets.

The prominent feature—scale-free property, manifested in the presence of multiple low-degree nodes and a few exceptionally important hubs, has been noted. The adjusted degree exponents *γ* for *Parkinson*, *Cancer I* and *Cancer II* belonged to the interval (2, 3), common for scale-free networks. The results were comparable to both literature-curated and experiment-based yeast interactomes, where *γ* oscillated between 2.1 and 2.5 (Yook *et al.*, 2004). On the other hand, for *HuRI γ* value exceeded three and the power-law fit matched only a small fraction of nodes.

The scale-free structure makes the networks immune to random perturbations and vulnerable to the targeted attacks. Such robustness examination may give insights into the networks topology. The differences in the level of robustness are possible and they are related to the *γ* value. The higher it is, the fewer connections the hubs have. This results in a more tight-knit structural pattern that demands the removal of a higher fraction of nodes, in order to decompose the network. Such behavior was noticed for *HuRI*, whose hubs were less impactful and hence *d_k_* and *d_B_* values were significantly higher. On the other hand, *d_k_* and *d_B_* values for *Parkinson*, *Cancer I* and *Cancer II* were relatively low when compared with those obtained in another study, for the yeast interactome, where $d_{k}=20\%,\;d_{B}=25\%$ (Iyer *et al.*, 2013). Another observation regards the smoothness of the curves generated by the robustness examination. At one extreme, there is *Parkinson* with multiple irregularities and jumps and on the other *HuRI* with all points smoothly decaying. Both behavior patterns were observed in other PPIN studies (Iyer *et al.*, 2013; Zotenko *et al.*, 2008). The differences may appear due to different data gathering procedures and, in consequence, different interactome sampling.

Another typical topological characteristic relates to the disassortative nature of PPINs, which was confirmed for *Parkinson*, *Cancer I* and *Cancer II*. The obtained degree correlation coefficients *r* values were slightly below zero, which is typical for both literature-based and experimental PPI data (Friedel and Zimmer, 2007; Iyer *et al.*, 2013). The rare assortative pattern, though still observable in Friedel and Zimmer (2007), where nodes of similar degree tend to choose one another, was recognized for *HuRI*. Such a positive *r* value influences the network decomposition process leading to an initial steeper decay for the betweenness centrality than for the degree (Iyer *et al.*, 2013) notable in *HuRI*.

The distributions of degree, betweenness centrality and eigenvector centrality in all networks were characterized by heavy tails. Nevertheless, a distinction between the tail lengths is notable, especially when comparing *Parkinson* with *Cancer I*, *Cancer II*, *HuRI* and other PPIN studies. For example, in Ran *et al.* (2013), its $C_{\text{max}},\;B_{\text{max}}$ and $k_{\text{rel}}$ values were similar to those obtained for *Cancers*, contrary to much higher values in *Parkinson*. The results for the *s*_1_ and *s*_2_ plots gave insights into the contribution of low- and high-degree nodes. In *HuRI*, we observed less impactful hubs and more middle-degree nodes, which stands in agreement with the central assumption of the uniform interactome sampling. In *Parkinson*, hubs were extremely influential and shortened the network’s distances, which is a consequence of the LRRK2 focus. *Cancer I* was located between *HuRI* and *Parkinson*, and *Cancer II* exhibited a tendency towards a *Parkinson* scheme. This shows that, although the number of data increased, its unbiased nature was negatively affected. In addition, the maximal values of centrality measures in *Cancer I* and *Cancer II* and other cancer’s PPIN studies (Chen *et al.*, 2016) correspond to different proteins. These observations indicate that the most important nodes are not necessarily the ones that are the most essential for the biological process. It may happen that those proteins which received the most attention during data gathering dominate. The level of scientific bias can be different, leading to dissimilar disturbances of the network model of the biological process.

In the failure cascade, a similar situation was noted. In the biological context, this algorithm can be seen as a procedure for examination of the effect that one malfunctioning protein is capable to induce in the organization of molecular processes. Concerning the simulation results for the networks, multiple nodes had low values of *P* and a few nodes were standing out of this pattern, having relatively very high *P*-values. Surprisingly, for *F *=* *0.25, not only the most influential nodes could induce high cascade effects but also other, less impactful nodes, had this property. However, it was only the case for *Parkinson* and *Cancer II*, characterized by strong interest biases. Such simulation outcomes uncovered that a stronger interest bias during the data gathering procedure leads to a multiple-level structural disturbance. This also shows that the temporal evolution of PPI datasets, as in *Cancers* is strongly biased by scientific interests. In consequence, the molecular interpretation of available models of biological systems is difficult to be performed.

*Parkinson*, *Cancer I* and *Cancer II* contain regions of high clusterization, which is detectable by high fractions of nodes with a clustering coefficient value around 1.0. In addition, the mean clustering coefficient value is similar to that obtained for the yeast interactome (Iyer *et al.*, 2013). Such observations stand in agreement with a theory of biological systems modularity (Yook *et al.*, 2004), according to which clustered regions correspond to functional modules. *HuRI* network significantly distinguishes from this pattern. Maybe the lower level of clusterization is sufficient to form functional modules. On the other hand, uniform sampling of the human interactome, a central assumption taken by the authors, may be the cause of such unusual behavior.

The PPINs of choice highly differ on the structural level when a detailed comparative analysis is performed. Although each of them seems to be somehow biased, *Cancer I* appeared to be the most neutral not revealing any special characteristics that would stand in disagreement with expected results. This network, although not the most certain and extensive one, seems to be appropriate for the analysis of molecular paths and retrieval of the most essential proteins. This shows that even if the interactions are experimentally less confident, the network may be structurally close to typical characteristics of PPINs. Therefore, the quality of the interactions themselves should not be the only criterion for the assessment of network usefulness. In addition, the bias evaluation should be performed before the biological analysis of the system. Should this step be ignored, misleading biological conclusions could be derived. For example, the drug target identification is often based on degree and betweenness centrality values. In *Parkinson*, such a procedure would lead to a strong bias towards the LRRK2 protein. We could also conclude that since 20% of the interactions come from two hubs, only these two proteins are essential for the disease. It may not correspond to reality, but may rather result from unusual attention to LRRK2. This protein also radically reduces distances in the network since many routes cross it. Given that network paths represent molecular mechanisms, their correct interpretation would be a more difficult task due to the LRRK2 focus. A similar situation would happen for *Cancer II*, which is more biased than *Cancer I*, although it contains more numerous, valuable data.

Another example of the problems generated by the data gathering procedure, which is often biased, regards the failure cascade. This simulation, due to its biological interpretation, could serve as an informative tool for the PPIN-based therapy design and disease understanding. Nevertheless, when analyzing the results for the PPINs of choice, it could be derived that different vulnerability of systems appears due to different biological mechanisms governing these networks. In networks that experience a big failure cascade one would want to target only these rare proteins, which have unexpectedly high propagation potential. Theoretically, their stimulation or blockade would impact the whole system. In consequence, our understanding of Parkinson’s disease and cancer would be highly disturbed. Similarly, the unusual nature of *HuRI* could lead to the conclusion that the overall interactome structure is different, meaning that it is governed by distinct dynamics of interactions. Nevertheless, in *HuRI*, the discrepancies probably happen due to a wider range of the study and lack of proteins not expressed in yeast. Such proteins could be the missing low-degree nodes attached to the hubs. Although the authors caution that the network is incomplete, it was not to be expected that it could lead to such unusual structural characteristics.

Network Science analyses of biological networks, such as those performed in this study, can be treated as a valuable source of the information regarding the global characteristics of molecular interactions. They can also serve to highlight possible methodological errors and biases introduced during the dataset construction. Not always generated topological results should be interpreted within a pure biological context because they are often affected by distribution of scientific interests. A consequence of such biases may be a highly disturbed model of the interactions. Due to inclusion of novel interactions, the temporal changes in an extended PPIN can, counter-intuitively, lead to a less reliable model of a biological process. A representative and uniform sampling of the interactome, as very difficult to be achieved, is still a Holy Grail of computational biology.

## Funding

This work was partially supported by the National Science Centre, Poland [2019/35/B/NZ2/03997] and Wroclaw Centre for Networking and Supercomputing at Wroclaw University of Science and Technology is acknowledged for the free access to computational resources.

*Conflict of Interest*: none declared.

## Supplementary Material

btac440_Supplementary_Data

## Data Availability

Data and code underlying this manuscript are available at https://github.com/AlicjaNowakowska/ETNA.
