# Correction to: Topological analysis as a tool for detection of abnormalities in protein–protein interaction data

**DOI:** 10.1093/bioinformatics/btac607

**Published:** 2022-09-08

This is a correction to: Alicja W Nowakowska, Malgorzata Kotulska, Topological analysis as a tool for detection of abnormalities in protein–protein interaction data, *Bioinformatics*, Volume 38, Issue 16, 15 August 2022, Pages 3968–3975, https://doi.org/10.1093/bioinformatics/btac440

In the originally published version of this manuscript the images for Figure 1 and Figure 2 were inadvertently swapped. Additionally in the fourth paragraph of the introduction two citations were incorrectly referenced as “study3, study4” after the sentence “Finally, it is even possible to try to predict new protein characteristics or functions on the basis of its network location, by combining the network analysis with other computational tools.” Additionally the header of the right hand column of table 3 included “(%)” in error.

These errors have now been corrected.

