# Supplementary material for: Topological analysis as a tool for detection of abnormalities in protein–protein interaction data
Source: Bioinformatics. 2022 Jun 30;38(16):3968–75. doi: 10.1093/bioinformatics/btac440 (PMC9746892; doi:10.1093/bioinformatics/btac440)
Supplement: btac440_Supplementary_Data [file btac440_supplementary_data.pdf]

Supplementary Materials

Alicja W. Nowakowska, Malgorzata Kotulska

Department of Biomedical Engineering, Faculty of Fundamental Problems of Technology, Wrocław University of Science and Technology, Wrocław, 50-370, Poland

1 Data

1.1 Downloading datasets from IntAct

The literature-curated datasets from IntAct were downloaded according to the following protocol:

1. Enter the IntAct website https://www.ebi.ac.uk/intact/home.
2. Go to *Download* tab and then *Curated Datasets* tab.
3. Click *Cancer/Parkinson.*
4. Choose *Select format to download* and then *XGMML Cytoscape.*

For HuRI the paper id - IM-25472 was inserted into the search box, then the list of interactions appeared and the file was downloaded by similarly choosing *Select format to Download* and then *XGMML Cytoscape.* The resulting files had .xml extension, which is not readable by graph-tool. Therefore, the format change was performed through Cytoscape and 'Save as' option. Manual deletion of special signs such as á, é, í, ü must have been done. Otherwise, graph-tool would give an error when reading a file. To fix this problem a text editor with an option 'search and replace' was used.

2 Software

2.1 Why graph-tool?

The engine for the network analysis within the created Extensive Tool for Network Analysis (ETNA) is based on **graph-tool** package (version 2.37) available in Python. Popular network libraries, such as Networkx or Igraph are slow when handling big datasets like the ones coming from high-quality protein interaction studies. Similarly, ready-to-use applications such as Gephi or Cytoscape, also commonly utilized, do not enable more advanced simulation coding. Graph-tool was constructed to deal with both problems, by building its computational architecture in C++, so that when calling built-in function the performance is comparable to pure C/C++ library. It enables rapid integration of the data with other general Python 3.6 possibilities.

2.2 Applied graph-tool functions

The following graph-tool functions constituted a programming workflow used for the network analysis:

- `graph_tool.load_graph(file_name.graphml)` - to load each network
- `MyNetwork.set_directed(False)` - to make networks' connections not directed
- `graph_tool.stats.remove_parallel_edges(MyNetwork)` - to remove parallel connections (repetitions)
- `MyNetwork.num_vertices()`, `MyNetwork.num_edges()` - to calculate number of nodes and connections in the networks

- `MyNetwork.edges()` and `MyNetwork.vertices()` - to iterate over the links and nodes of the networks and `MyNetwork.properties[("e"/"v","attribute")][LinkNumber/NodeNumber]` to extract nodes' and links' attributes values. Later, frequently such information is utilized as an input to Python's **pandas** library's DataFrame function (`pd.DataFrame({MetricName:list_of_values})`) to give statistics on how many times each attribute value is assigned (`MyDataFrame[MetricName].value_counts()`).

- `graph_tool.topology.label_components(MyNetwork)` - to extract information about a number and population of networks' components

- `graph_tool.label_largest_component(MyNetwork)` and `MyNetwork.remove_vertex(NodeNumber)` - to extract the largest connected component of the networks. Each node in the network is labelled according to the network component that it belongs to. All the nodes not included in the largest connected component are removed.

- `MyNetwork.degree_property_map("total")` - to obtain a map NodeNumber : corresponding degree

- `graph_tool.centrality.betweenness(MyNetwork)` - to obtain a map NodeNumber : corresponding betweenness centrality value

- `graph_tool.centrality.closeness(MyNetwork)` - to obtain a map NodeNumber : corresponding closeness centrality value

- `graph_tool.centrality.eigenvector(MyNetwork)` - to obtain a map NodeNumber : corresponding eigenvector centrality value

- `graph_tool.clustering.local_clustering(MyNetwork)` - to obtain a map NodeNumber : corresponding clustering coefficient value

- `graph_tool.topology.shortest_distance(MyNetwork,NodeNumber)` - to extract a list of all lengths of the shortest paths starting in a node with NodeNumber

- `graph_tool.assortativity(MyNetwork,"total")` - to calculate assortativity coefficient value for the networks

- `graph_tool.avg_neighbor_corr(MyNetwork,"total","total")` - to retrieve information on average degree of the nearest neighbours

In what regards the outputs of the map form (centrality and clustering metrics functions), to obtain a list of the measure's values one needs to apply .fa attribute (`results.fa`).

2.3 Other libraries

The power law fitting using maximum likelihood was done using R package **poweRlaw**. The results were integrated with Python code using **rpy2** library.

To perform robustness examination in terms of random failures **numpy.random.shuffle** from Python's **numpy** library was used.

Plots were prepared using **matplotlib** and **seaborn** library in Python.

The computational workflow was integrated into ETNA using Python's **ipywidgets** library. Python's **pandas** library was extensively used for saving and analyzing the data. The specific functions and their applications were the following:

- `pd.DataFrame({FirstMetric:ListOfValues,SecondMetric:ListOfValues,...})` - to create DataFrames with the results

- `MyDataFrame.sort_values(by=MetricName,ascending=False)` - to sort records according to the metric

- `MyDataFrame.head(n=AppropriateNumber)` - to identify decomposition fraction and top nodes

- `MyDataFrame.to_csv()` - to convert DataFrame into the csv format

2.4 How to use ETNA?

2.4.1 Option 1. Jupyter notebook on your computer

To run ETNA you must fulfill the following criteria:

- Have an .ipynb interpreter programme e.g. Jupyter Notebook application that can be installed within Anaconda environment.
- Install graph-tool library. The instructions can be found on the website: https://git.skewed.de/count0/graph-tool/-/wikis/installation-instructions.
- Install all other libraries: numpy, pandas, random, rpy2, ipywidgets, base64, hashlib, typing and seaborn. If you have a Jupyter notebook you may install a library by typing in the notebook's cell `!pip install LibraryName`.

Option 1.1. When the installation is done go to the GitHub repository https://github.com/AlicjaNowakowska/ETNA. Download an .ipynb file called `ETNAYourOwnJupyterInterpreter.ipynb`. After the installation, open the downloaded notebook and run all the cells by clicking Shift+Enter buttons. After running the last cell ETNA's window will show up.

Option 1.2. Alternatively, you may download `ETNA_source_code2.py` file to your working directory. Open a new Jupyter notebook, type in a cell `%run -i ETNA_source_code2.py` and run it by clicking Shift+Enter buttons. The ETNA's window will show up.

If you encounter that your ETNA's window is too small, type at the beginning of the cell the following:

```
from IPython.core.display import display, HTML
display(HTML("<style>.container { width:100% !important; }</style>"))
display(HTML("<style>.container { height:100% !important; }</style>"))
display(HTML("<style>.output_result { max-width:100% !important; }</style>"))
display(HTML("<style>.output_result { max-height:100% !important; }</style>"))
```

2.4.2 Option 2. Google Colab

You may run ETNA using online Google Colab application that requires a free gmail account. Log in to your gmail account. Go to the page:
https://githubtocolab.com/AlicjaNowakowska/ETNA/blob/main/ETNAColab.ipynb The colab notebook will appear. Run each code cell by clicking Shift+Enter button. After running the last cell ETNA's window will show up.

2.4.3 Working with ETNA

When ETNA's window shows up you can enjoy the network analysis tool. The four (preprocessed - special signs removed and file formats transformed to .graphml) datasets analyzed in the paper are provided in the Github repository in ExemplaryData.zip file.

To perform a network analysis using ETNA you need to provide a file path in the text box (`Provide file path here`). After providing the file path you must prepare the network for the analysis by hitting `Prepare the network` button. Now, choose the method of interest by clicking the corresponding tab in the ETNA's window. Adjust the settings if present and hit `Run` button. The results and plots will appear on the right. If you want to perform the analysis for other network hit `Restart GUI` button located at the bottom of the window. The additional instructions are also provided in the interface.

3 Additional notes on methods and results

3.1 IntAct MI score distribution

The violin plots for the datasets' IntAct MI score distribution are presented in Figure S1

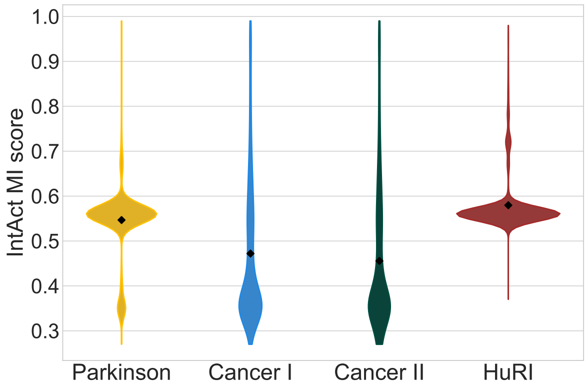

Fig. S1: IntAct MI score distribution presented in the violin plot. Diamond denotes the mean.

3.2 Histograms of topological measures

The discussed distributions of the degree, betweenness centrality, closeness centrality, eigenvector centrality and clustering coefficient are presented in the form of the histograms in Figure S2, Figure S3, Figure S4, Figure S5, Figure S6 respectively.

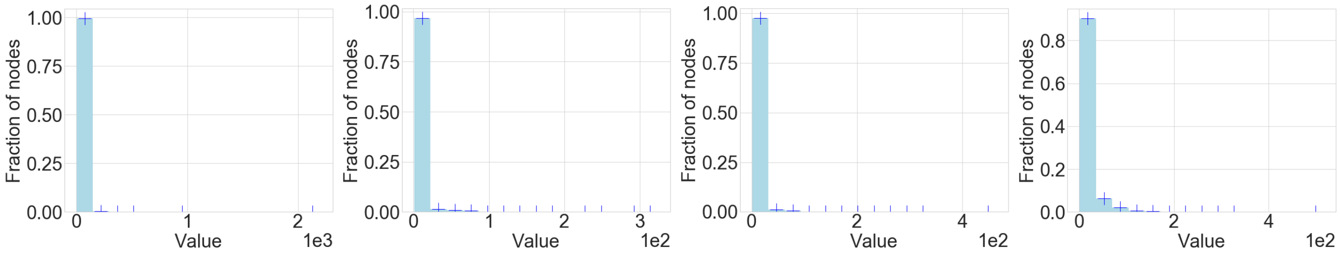

(a) *Parkinson* (b) *Cancer I* (c) *Cancer II* (d) *HuRI*

Fig. S2: Degree histograms

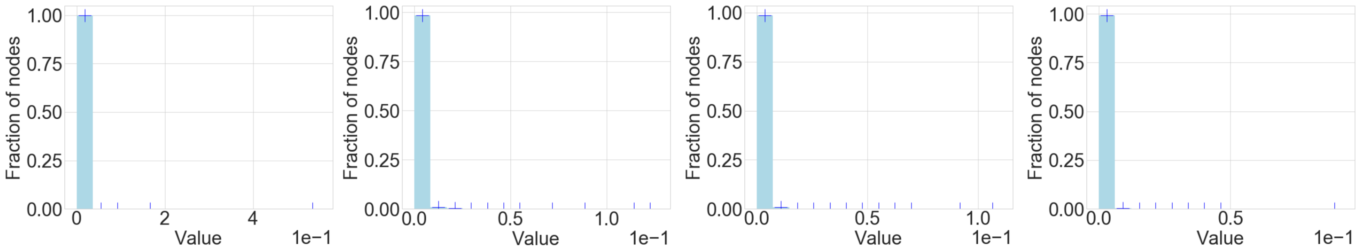

(a) *Parkinson* (b) *Cancer I* (c) *Cancer II* (d) *HuRI*

Fig. S3: Betweenness centrality histograms

3.3 Power law fitting

The details of the power law adjustment to the degree distribution are discussed in this section. Degree sequence is understood as the vector $(k_1, k_2, \ldots, k_n)$, where k_i is degree of an ith node.

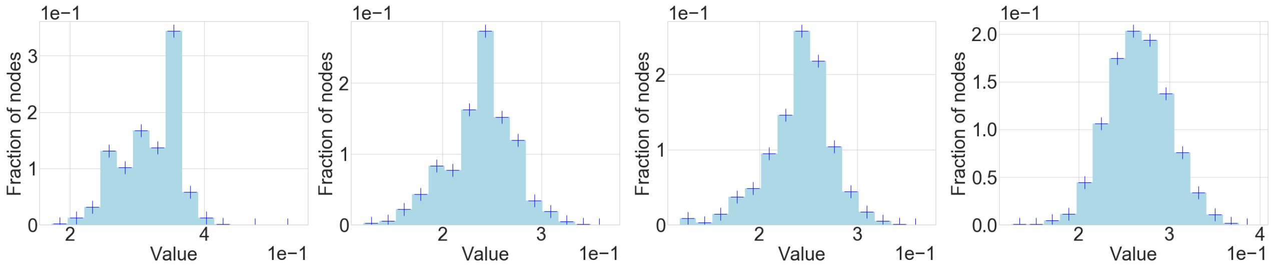

(a) *Parkinson* (b) *Cancer I* (c) *Cancer II* (d) *HuRI*

Fig. S4: Closeness centrality histograms

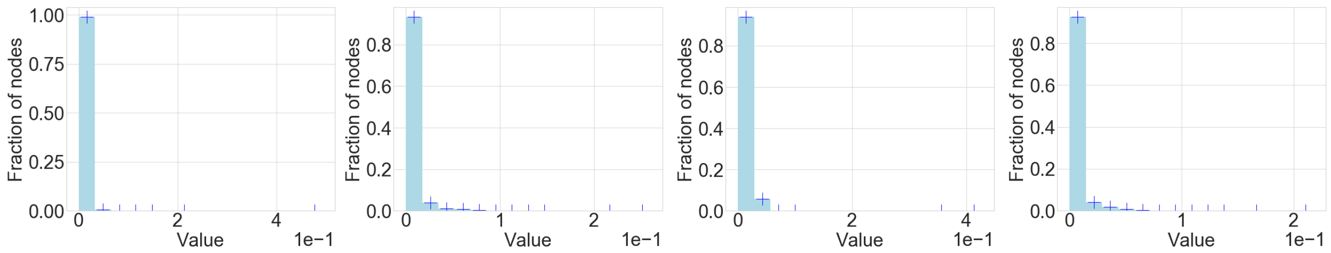

(a) *Parkinson* (b) *Cancer I* (c) *Cancer II* (d) *HuRI*

Fig. S5: Eigenvector centrality histograms

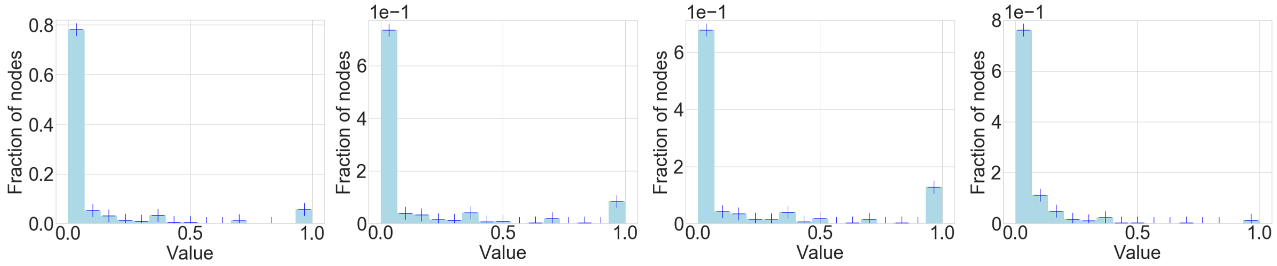

(a) *Parkinson* (b) *Cancer I* (c) *Cancer II* (d) *HuRI*

Fig. S6: Clustering coefficient histograms

3.3.1 Power law distribution

The power law distribution definition is the following:

$$p_k = Ck^{\gamma}. \tag{1}$$

Usually, $2 < \gamma < 3$. The values below 2 are considered as anomalous. Those in the common regime cause the variance of the degree distribution to go to infinity when the network size does it. Such γ guarantees *ultra-small world* property - hubs drastically reduce the lengths of the paths by linking to multiple low-degree nodes. If the value of 3 is exceeded, second moment is now finite and the impact of hubs is not so evident.

3.3.2 Maximum likelihood estimator and k_{min}

The maximum likelihood method assumes existence of some cutoff degree k_{min} - point from which the data comes from the power law distribution. It is because, frequently, only the tail of the sorted increasingly degree sequence can be described in this way. The probability of appearance of a node of degree k is formalized as [1]:

$$p_k = \frac{k^{-\gamma}}{\zeta(\gamma, k_{\mathrm{min}})} \tag{2}$$

where:

$$\zeta(\gamma, k_{\min}) = \sum_{n=0}^{\infty}(n + k_{\min})^{-\gamma}. \tag{3}$$

The approximation of the maximum likelihood estimator implemented in R package poweRlaw that was used is the following:

$$\hat{\gamma} = 1 + n(\sum_{i=1}^{n} \ln \frac{k_i}{k_{\min} - 0.5}). \tag{4}$$

The cutoff parameter k_{min} was to be found as a one that minimizes the following:

$$D = max_{x \geq k_{\min}} |S(x) - P(x)| \tag{5}$$

where $S(x)$ and $P(x)$ are the Cumulative Distribution Functions ($P(X < k)$, denoted as CDFs of the data and model respectively (for $x \geq k_{\min}$).

3.3.3 Statistical test for the fit

The following were the testing hypotheses:
H_0 : network degree distribution follows a power law with given γ and $k_{\min}$.
H_1 : network degree distribution does not follow a power law with given γ and $k_{\min}$.

A bootstrapping procedure was used to perform the test. Data from the theoretical distribution is generated B times and a distance D_{sim} to the exact CDF was measured for each sample. Next, it was calculated how many times D_d for the degrees dataset was larger than D_{sim} for the simulated data on this basis associating a p-value result [1] for the test.

3.3.4 Non-optimal fit

According to the described procedure, $\hat{\gamma}$ and $k_{\min}$ were adjusted to the networks degree distributions. In the *Parkinson* network γ was estimated to be 2.16 with a cutoff at $k_{\min} = 8$. In *Cancer I* $\gamma = 2.16$ and $k_{\min} = 4$ and in *Cancer II* $\gamma = 2.19$, $k_{\min} = 3$. In *HuRI* a giant part of the nodes must be discarded to fit γ whose value is 3.59 with $k_{\min} = 90$. The obtained p-values calculated using package's bootstrap function with B equal 500 were 0.046, 0.12, 0.03 and 0.47 for *Parkinson*, *Cancer I*, *Cancer II* and *HuRI*, respectively.

3.3.5 Methodology for finding optimal fit

We noted that with such an approach we discard many nodes in *HuRI.* The p-value for Parkinson was very low and leads to the rejection of H_0. Therefore, a modified approach for $k_{\min}$ finding was applied. Instead of using a built-in procedure for its optimization as previously, this time an option of fixing $k_{\min}$ value and then finding the corresponding γ with ML method was utilized. Different $k_{\min}$ values were checked until the p-value of the test exceeded 0.1 guaranteeing a secure base for non-rejection of the power law hypothesis (Algorithm 1).

Algorithm 1 Finding optimal $k_{\min}$

```
k_min = 1, p-value = 0
while p-value < 0.1 do
    find γ using ML method
    calculate p-value of the test
    if p-value> 0.1 then
        BREAK the loop
    else
        k_min = k_min + 1
    end if
end while
return k_min and γ
```

In this way it was possible to find the lowest possible secure $k_{\min}$ leading to discarding as little data as possible. The relation between calculated from bootstrapping p-value and $k_{\min}$ for each network is shown in Figure S7.

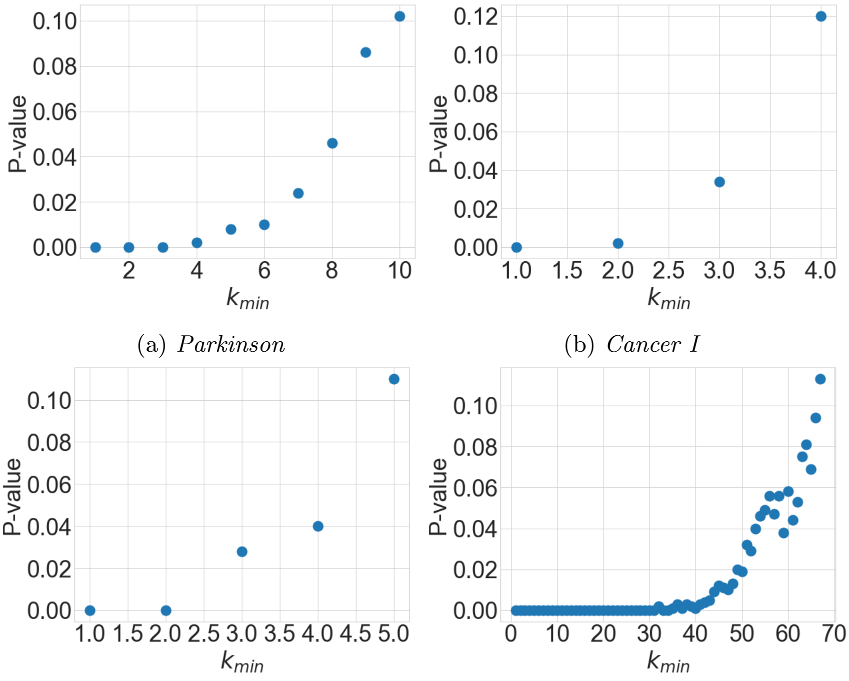

(a) *Parkinson* (b) *Cancer I*

(c) *Cancer II* (d) *HuRI*

Fig. S7: Power law hypothesis

3.3.6 Final and optimal fit results

The final results of the maximum likelihood fitting for all the networks are presented in Figure S8. This fit was presented in the paper.

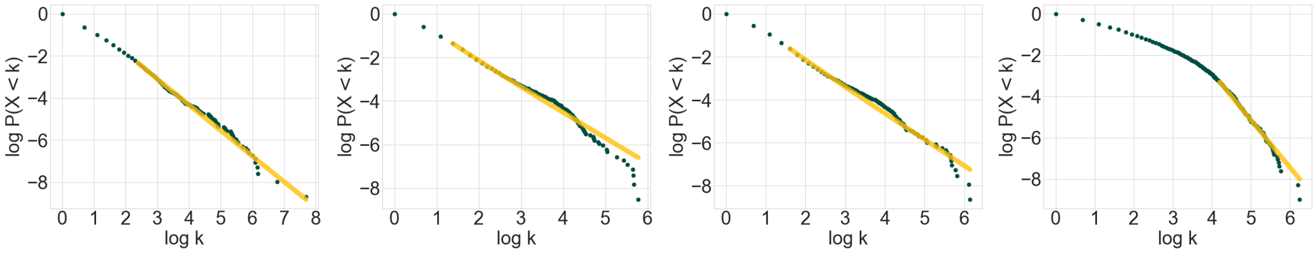

(a) *Parkinson* (b) *Cancer I* (c) *Cancer II* (d) *HuRI*

Fig. S8: Final maximum likelihood fit

3.4 Robustness examination

3.4.1 Algorithm

The algorithms used for robustness examination were the following:

Algorithm 2 Algorithm for the robustness assessment with respect the random failures

$fraction = 0$, MC is the number of Monte Carlo repetitions performed, $step$
while $fraction < 1.0$ **do**
  for i=1:MC **do**
    Initialize the network, N is the number of nodes it has, shuffle the nodes order
    Remove $fraction \cdot N$ of the top nodes
    Calculate the size (number of nodes) of the largest connected component of the network
  end for
  Average the largest connected component size for the fraction over MC repetitions
  $fraction = fraction + step$
end while
Return array of pairs (fraction:average largest connected component size)

Algorithm 3 Algorithm for the robustness assessment with respect the attacks

$fraction = 0$, sort nodes by the centrality measure, $step$
while $fraction < 1.0$ **do**
  Initialize the network, N is the number of nodes it has
  Remove $fraction \cdot N$ of the top nodes
  Calculate the size (number of nodes) of the largest connected component of the network
  $fraction = fraction + step$
end while
Return array of pairs (fraction:largest connected component size)

3.4.2 Results for *Parkinson* and *Cancer I*

The plots for *Cancer I* and *Cancer II* are presented in Figure S13. The plots for *Parkinson* and *HuRI* were presented in the paper.

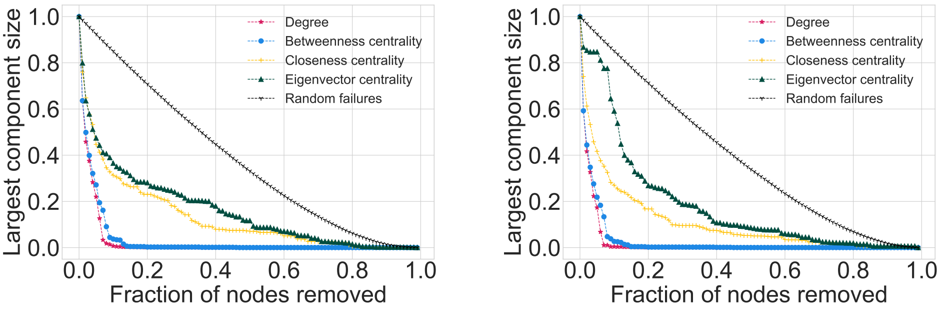

(a) *Cancer I*   (b) *Cancer II*

Fig. S9: Results of the robustness measurements

3.5 Failure cascade simulation results

3.5.1 Algorithm

The algorithm of the failure cascade was the following:

Algorithm 4 Algorithm for the failure cascade simulation

failure fraction F, GO = TRUE, provide the initial node n
set the status of n to *failed*, the rest of the nodes have the status *not failed*
while GO = TRUE **do**
  GO = False
  for each node v from the network with the status *not failed* **do**
    extract statutes of the neighbours of the node v
    if more than $F\cdot$number of neighbours have a status of *failed* **then**
      change status of v to *failed*
      GO = TRUE
    end if
  end for
end while
Return P - a final percentage of nodes with the status *failed*

3.5.2 Distribution of the failure cascade size

The failure cascade results for $F = 0.25$, $F = 0.5$ and $F = 0.75$ are presented in Figure S10, Figure S11, Figure S12 respectively. The plots show what fractions of nodes generated certain value of the cascade size.

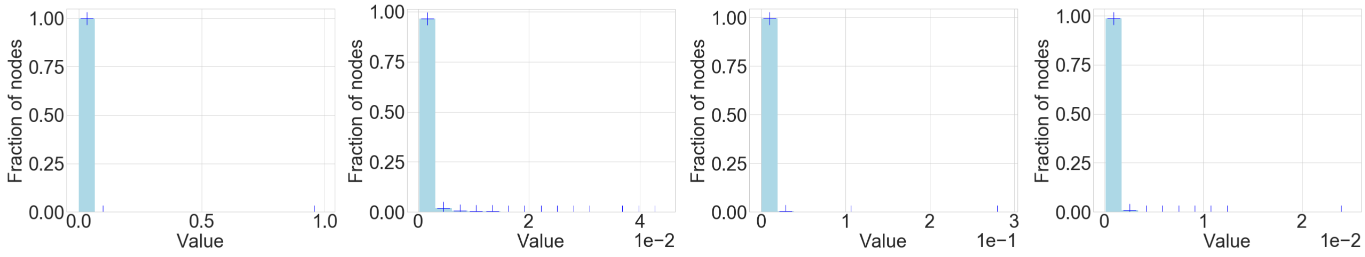

(a) *Parkinson* (b) *Cancer I* (c) *Cancer II* (d) *HuRI*

Fig. S10: Failure cascade sizes distribution for $F = 0.25$

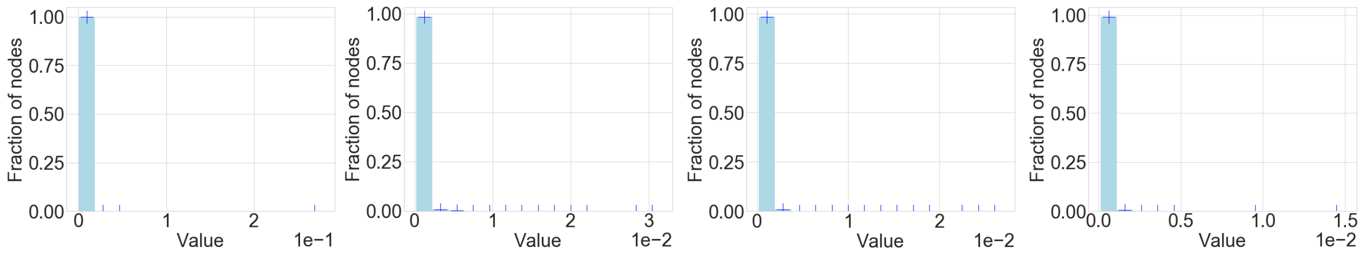

(a) *Parkinson* (b) *Cancer I* (c) *Cancer II* (d) *HuRI*

Fig. S11: Failure cascade sizes distribution for $F = 0.5$

3.5.3 Comparison of the failure cascade with other measures

The failure cascade simulation seems to be a promising tool for the evaluation of the nodes' propagation potential and in consequence may help in drug target identification. To check whether it indeed cannot be replaced by other metrics, we calculated the Pearson correlation coefficient values between the cascade size and other metrics (degree, betweenness centrality, closeness centrality, eigenvector centrality and clustering coefficient) for each network. The results are presented in Figure S13.

The failure cascade in none of the cases could be perfectly described by any other measure. Nevertheless, the significant correlations between cascade sizes and degree and betweenness centrality were noted. The clustering coefficient and closeness centrality are poor indicators for the cascade size. The lowest correlation values are

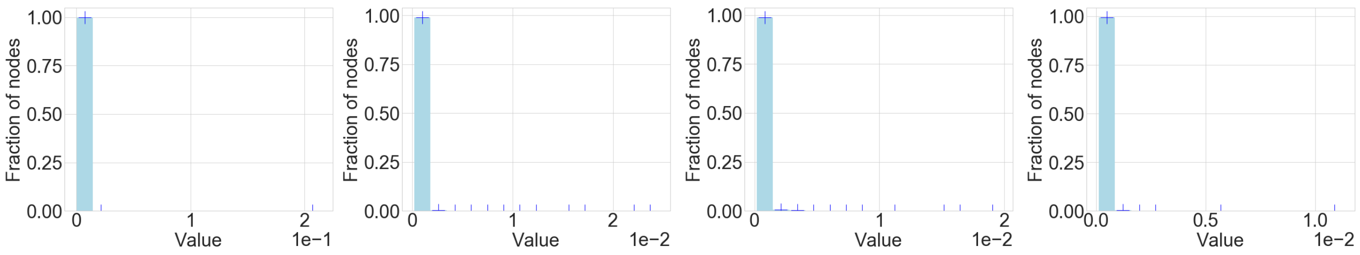

(a) *Parkinson* (b) *Cancer I* (c) *Cancer II* (d) *HuRI*

Fig. S12: Failure cascade sizes distribution for $F = 0.75$

usually obtained for the row corresponding to cascade sizes from $F = 0.25$ simulation, though *HuRI* is an exception from this trend. The results cascade sizes $F = 0.5$ and cascade sizes $F = 0.75$ have high correlation values indicating that they provide similar results. The correlation values between cascade sizes $F = 0.25$ and cascade sizes $F = 0.5$ and $F = 0.75$ are much lower.

These observations show that the results for the failure cascade are unique and useful since they are network-specific. They cannot be predicted by other measures and the lower F values tend to highlight other nodes that do not seem to be important from other metrics' perspectives. The molecular interpretation of the F parameter is hard to be performed. Therefore, it would be recommended to experimentally estimate neighbours fraction threshold that causes the error transmission. In other words, how many protein interactors must malfunction to make the protein of choice malfunctioning too. This would make the simulation to model even better the real processes.

4 Proposed values indicating that your network is biased

Based on our analyses below we propose intervals for different metrics which may suggest a biased network structure due to scientific interests.

Metric	Proposed values suggesting biased system
Degree correlation coefficient	>0 (assortative structure)
Relative maximal degree value ($k_{\max}/N$)	>0.15 (very influencing hub)
Highest betweenness centr. ($B_{\max}$)	>0.2 (very influencing bottleneck)
Decomposition fraction for degree d_k	>0.15 (highly robust network)
Decomposition fraction for betweenness centr. d_B	>0.2 (highly robust network)
Failure cascade maximal value $P_{\max}$ for $F = 0.25$	>20%

	Degree	Betweenness centrality	Closeness centrality	Eigenvector centrality	Clustering coefficient	Cascade size F=0.25	Cascade size F=0.5	Cascade size F=0.75
Degree	1.000000	0.915612	0.173730	0.859421	-0.024388	0.656704	0.881889	0.847629
Betweenness centrality	0.915612	1.000000	0.117610	0.738880	-0.013746	0.718184	0.990448	0.979041
Closeness centrality	0.173730	0.117610	1.000000	0.564594	0.272597	0.078397	0.096116	0.087823
Eigenvector centrality	0.859421	0.738880	0.564594	1.000000	0.101371	0.514342	0.695082	0.664396
Clustering coefficient	-0.024388	-0.013746	0.272597	0.101371	1.000000	-0.010054	-0.012248	-0.010278
Cascade size F=0.25	0.656704	0.718184	0.078397	0.514342	-0.010054	1.000000	0.712254	0.708207
Cascade size F=0.5	0.881889	0.990448	0.096116	0.695082	-0.012248	0.712254	1.000000	0.995365
Cascade size F=0.75	0.847629	0.979041	0.087823	0.664396	-0.010278	0.708207	0.995365	1.000000

(a) *Parkinson*

	Degree	Betweenness centrality	Closeness centrality	Eigenvector centrality	Clustering coefficient	Cascade size F=0.25	Cascade size F=0.5	Cascade size F=0.75
Degree	1.000000	0.917178	0.377157	0.802116	-0.034054	0.843965	0.891261	0.821663
Betweenness centrality	0.917178	1.000000	0.282168	0.692722	-0.049745	0.822898	0.913250	0.873893
Closeness centrality	0.377157	0.282168	1.000000	0.614549	0.000100	0.188534	0.209876	0.188432
Eigenvector centrality	0.802116	0.692722	0.614549	1.000000	-0.042865	0.503932	0.558431	0.515078
Clustering coefficient	-0.034054	-0.049745	0.000100	-0.042865	1.000000	-0.031709	-0.046300	-0.045042
Cascade size F=0.25	0.843965	0.822898	0.188534	0.503932	-0.031709	1.000000	0.871699	0.789997
Cascade size F=0.5	0.891261	0.913250	0.209876	0.558431	-0.046300	0.871699	1.000000	0.960981
Cascade size F=0.75	0.821663	0.873893	0.188432	0.515078	-0.045042	0.789997	0.960981	1.000000

(b) *Cancer I*

	Degree	Betweenness centrality	Closeness centrality	Eigenvector centrality	Clustering coefficient	Cascade size F=0.25	Cascade size F=0.5	Cascade size F=0.75
Degree	1.000000	0.904337	0.342386	0.578659	-0.045974	0.609409	0.856866	0.711182
Betweenness centrality	0.904337	1.000000	0.275384	0.425388	-0.061818	0.554068	0.910157	0.824172
Closeness centrality	0.342386	0.275384	1.000000	0.328542	0.090692	0.115856	0.208198	0.184566
Eigenvector centrality	0.578659	0.425388	0.328542	1.000000	0.268712	0.314994	0.317110	0.163754
Clustering coefficient	-0.045974	-0.061818	0.090692	0.268712	1.000000	-0.028901	-0.059900	-0.056549
Cascade size F=0.25	0.609409	0.554068	0.115856	0.314994	-0.028901	1.000000	0.626692	0.423974
Cascade size F=0.5	0.856866	0.910157	0.208198	0.317110	-0.059900	0.626692	1.000000	0.926690
Cascade size F=0.75	0.711182	0.824172	0.184566	0.163754	-0.056549	0.423974	0.926690	1.000000

(c) *Cancer II*

	Degree	Betweenness centrality	Closeness centrality	Eigenvector centrality	Clustering coefficient	Cascade size F=0.25	Cascade size F=0.5	Cascade size F=0.75
Degree	1.000000	0.767064	0.601658	0.801317	0.032730	0.673100	0.594249	0.488290
Betweenness centrality	0.767064	1.000000	0.344330	0.533104	-0.022827	0.875255	0.838766	0.792602
Closeness centrality	0.601658	0.344330	1.000000	0.614203	0.174879	0.297627	0.257206	0.209177
Eigenvector centrality	0.801317	0.533104	0.614203	1.000000	0.131238	0.414474	0.351857	0.262338
Clustering coefficient	0.032730	-0.022827	0.174879	0.131238	1.000000	-0.046917	-0.040565	-0.034431
Cascade size F=0.25	0.673100	0.875255	0.297627	0.414474	-0.046917	1.000000	0.927723	0.856397
Cascade size F=0.5	0.594249	0.838766	0.257206	0.351857	-0.040565	0.927723	1.000000	0.942143
Cascade size F=0.75	0.488290	0.792602	0.209177	0.262338	-0.034431	0.856397	0.942143	1.000000

(d) *HuRI*

Fig. S13: Pearson's correlation coefficients values between the failure cascade sizes and other metrics. Colors denote the heatmap.

References

[1] Colin S. Gillespie. Fitting heavy tailed distributions: the powerlaw package. 2014.
